# A retrospective observational study on the safety and efficacy of first-line treatment with bevacizumab combined with FOLFIRI in metastatic colorectal cancer

**DOI:** 10.1038/sj.bjc.6605938

**Published:** 2010-10-12

**Authors:** R López, M Salgado, M Reboredo, C Grande, J C Méndez, M Jorge, C Romero, G Quintero, J de la Cámara, S Candamio

**Affiliations:** 1 Department of Oncology, Complejo Hospitalario Universitario de Santiago, Travesía da Choupana, s/n, 15706 Santiago de Compostela, Spain; 2 Department of Oncology, Complejo Hospitalario de Ourense, Ramón Puga, 54, 32005 Orense, Spain; 3 Department of Oncology, Complejo Hospitalario Universitario A Coruña, Xubias de Arriba, 84, 15006 A Coruña, Spain; 4 Department of Oncology, Hospital Meixoeiro, Camino de Meixueiro, s/n, 36214 Vigo, Spain; 5 Department of Oncology, Centro Oncológico de Galicia, Avenida de Montserrat, s/n, 15009 A Coruña, Spain; 6 Department of Oncology, Hospital Xeral Cíes, Calle de Pizarro, 2, 36204 Vigo, Spain; 7 Department of Oncology, Hospital Provisa, Calle de Salamanca, 5, 36211 Vigo, Spain; 8 Department of Oncology, Complejo Hospitalario Xeral Calde, Doctor Severo Ochoa, s/n, 27004, Lugo, Spain; 9 Department of Oncology, Hospital Arquitecto Marcide, Carretera de San Pedro de Leixa, s/n, 15405 Ferrol, Spain

**Keywords:** colorectal cancer, bevacizumab, FOLFIRI, first-line treatment

## Abstract

**Background:**

Combination of bevacizumab and FOLFIRI has currently become one of the standard therapeutic regimens. However, published information is still limited. The objective of the present retrospective observational study is to analyse the response and toxicity of first-line treatment with FOLFIRI+bevacizumab in patients with metastatic colorectal cancer (mCRC).

**Methods:**

Data were collected from patients from nine Spanish sites diagnosed with mCRC, ECOG⩽2, whose first treatment for advanced disease was at least three cycles of FOLFIRI+bevacizumab.

**Results:**

A total of 95 patients were enrolled into the study: 64.2% males, median age of 59 years (53.2–67.1 years), ECOG=0–1 in 96.9% of patients. The main site of primary tumour was the colon (69.7%), and most metastases occurred in the liver (71.6%). Clinical benefit was detected in 67.4% (57.0–76.6; 95% confidence interval (CI)), with 8.4% of CR and 42.1% of PR. Median TTP was 10.6 months (10.0–11.3; 95% CI), PFS was 10.6 months (9.8–11.3; 95% CI), and OS was 20.7 months (17.1–24.2; 95% CI). Main grade I–II toxicities included haematological toxicity (35.8%), diarrhea (27.3%), mucositis (25.3%), asthenia (19.0%), haemorrhages (11.6%), and emesis (10.6%). Toxicities reaching grades III–IV were haematological toxicity (9.5%), diarrhea (8.5%), mucositis (5.3%), hepatic toxicity (2.1%), asthenia (2.1%), proteinuria (1.1%), emesis (1.1%), pain (1.1%), and colics (1.1%).

**Conclusion:**

Results of this study support the beneficial effect of adding bevacizumab to FOLFIRI regimen in terms of efficacy and show a favourable tolerability profile.

Colorectal cancer (CRC) is the third most common malignancy, and the fourth cause of death by cancer worldwide. The World Health Organization (WHO) estimates an incidence of 945 000 new cases yearly and 492 000 deaths (WHO, 2003). Colorectal cancer is considered the second most important cancer location; it has a growing trend of 2.6% per year and a higher mortality in males (López *et al*, 2004). Surgery is the main curative treatment in early stages. Moreover, a high proportion of patients develop metastases, which is considered the leading cause of treatment failure and death (Fortner, 2007).

For >40 years, standard treatment of patients with metastatic CRC (mCRC) has been 5-fluorouracil (5-FU); various strategies have been developed to maximise its cytotoxic effect, including dose modifications, dosage, and route of administration (Meta-analysis Group in Cancer, 1998). The concurrent administration of the biochemical modulator leucovorin (folinic acid, LV) has improved the efficacy of 5-FU (Thirion *et al*, 2004). Furthermore, the combination of irinotecan with the 5-FU+LV regimen, in bolus or intravenous infusion (FOLFIRI), has been shown to improve the rate of response and prolong the progression-free survival (PFS) and overall survival (OS) (Douillard *et al*, 2000; Saltz *et al*, 2000; Kohne *et al*, 2005). The results obtained with this regimen have made it the reference treatment, replacing 5-FU+LV, and permitted to obtain the United States Food and Drug Administration (FDA) and the European Medicines Agency (EMA) approvals for irinotecan in combination with 5-FU+LV, in bolus and in infusion, as first-line treatment of mCRC. The combination of 5-FU+LV with oxaliplatin (FOLFOX), developed by the French group, has an activity similar to FOLFIRI. Either of these two schemes is considered the standard treatment in first- and second-line mCRC.

Bevacizumab is a humanised monoclonal antibody that acts binding to and inhibiting the action of vascular endothelial growth factor (VEGF) (Presta *et al*, 1997). Clinical studies have provided a wide evidence of its efficacy and safety in combination with various chemotherapy regimens for the treatment of CRC (Kabbinavar *et al*, 2003, 2005; Hurwitz, 2004; Hurwitz *et al*, 2005; Hochster, 2006), and it has even been considered the only anti-angiogenic agent that, combined with chemotherapy, has shown to prolong survival in patients with mCRC (Diaz-Rubio and Schmoll, 2005). This chemotherapy potentiating effect may be associated with bevacizumab's effect of reducing tumoural perfusion, vascular volume, microvascular density, and interstitial fluid pressure. These effects permit the normalisation of the tumour pressure and permeability, thus facilitating the access of chemotherapy agents to the cancer cells (Willett *et al*, 2004). Additionally, bevacizumab (Avastin) has been approved by the FDA and EMA as first-line treatment of mCRC in combination with intravenous regimens of 5-FU+LV with or without irinotecan. One of the most used regimens with irinotecan is the combination of bevacizumab with FOLFIRI. However, even though this combination has become one of the standard therapeutic regimens, published information to date is still limited. Therefore, this study intends to expand the current available knowledge, and its main objective consists of assessing the rate of response to the administration of bevacizumab in combination with FOLFIRI as first-line treatment for mCRC.

## Materials and methods

### Selection criteria

Male and female patients ⩾18 years of age, with an Eastern Cooperative Oncology Group (ECOG) status ⩽2, histological diagnosis of advanced (locally advanced or metastatic, non-resectable) measurable CRC, according to the Response Evaluation Criteria in Solid Tumours (RECIST). All patients must have had received at least three cycles of FOLFIRI+bevacizumab as their first treatment for advanced disease. For patients who had received prior radiation therapy, the target lesions were selected among those not irradiated, unless progression of these lesions into the irradiated field was documented.

### Study design and treatment

The primary objective of this multicentre, retrospective, observational study is to assess the overall response (OR) rate obtained in patients with locally advanced or metastatic non-resectable CRC treated with FOLFIRI+bevacizumab. The secondary objectives of this study were to assess the progression-free interval and the safety profile of the cancer treatment. In order to identify the information needed to achieve these objectives, oncologists from nine sites in Spain reviewed the clinical records of 95 patients treated with at least three cycles of FOLFIRI+bevacizumab during the previous year.

### Assessment of efficacy

The assessment of the efficacy of the FOLFIRI+bevacizumab combination was done by the investigators according to the RECIST criteria. All patients had one CT at baseline, after the third cycle and, then, every three cycles or progression of the disease. Additionally, the time to progression (TTP), the PFS, and the OS were assessed.

### Assessment of safety

The safety of the FOLFIRI and bevacizumab combination was assessed based on data collected from the medical charts about the number and kind of toxicities experienced by the patients included in the study. The severity of the toxicities was established according to the toxicity criteria of the National Cancer Institute (NCI-CTC).

### Statistical and analytical methods

The main variable is the rate of response according to the RECIST criteria, expressed as absolute frequency (*n*), relative frequency (%), and the confidence interval (CI) at 95% of the rates of complete, partial, and OR. In the survival analyses, the following parameters were included: OS (time between the first treatment administration and exitus for any cause), TTP (time between the first treatment administration until progression or death due to progression), and PFS (time between initiation of treatment until progression or death due to any cause). These analyses were done with the Kaplan–Meier method, with a CI of 95%. The safety results obtained from the population included in the study are expressed as absolute and relative frequencies of each kind of adverse event. The statistical analysis was done with the Statistical Package of the Social Sciences (SPSS), version 17.0.

This study has been conducted in accordance with the directives established by the Good Clinical Practices directives and the Declaration of Helsinki. The study protocol has been approved by the ethics committees, and all the patients have given their informed consent to participate in the study.

## Results

**Figure 1:**
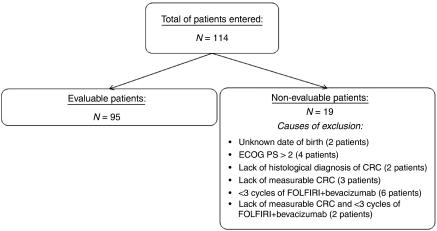
Flowchart of patients entered into the study.

A total of 114 patients were consecutively treated with FOLFIRI+bevacizumab in the Departments of Oncology of nine sites in Galicia (Spain) from March 2005 to November 2007, 95 of them fulfilled the inclusion/exclusion criteria (Figure 1). Table 1 shows the characteristics of the patients at the baseline visit. The median age was 59 years (range: 53.2–67.1 years), and 96.9% of patients had an ECOG status 0–1. The most common location of the primary tumour was the colon (69.7%), whereas the liver was the most common metastatic site (71.6%).

### Assessment of efficacy

The OR (complete response (CR)+partial response (PR)) was 50.5% (40.1–60.9; 95% CI) of patients, with CR in 8.4% of cases (3.7–15.9; 95% CI) (Table 2). Moreover, 67.4% (57.0–76.6; 95% CI) of patients obtained clinical benefit (CR+PR+stable disease (SD)).

**Figure 2:**
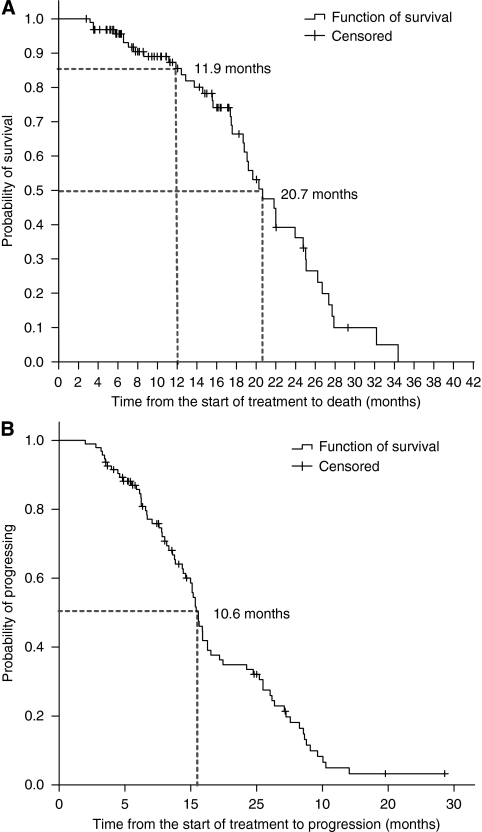
Kaplan–Meier estimates of survival curves: overall survival (*N*=95) (**A**) and progression-free survival (*N*=94) (**B**).

The median OS of patients in the study was 20.7 months (17.1–24.2; 95% CI), the median TTP was 10.6 months (10.0–11.3; 95% CI), and the median PFS was 10.6 months (9.8–11.3; 95% CI) (Figure 2).

### Assessment of safety

The most frequent toxicities throughout the study were the following: haematological toxicity (45.3%), diarrhea (35.8%), mucositis (30.5%), asthenia (21.1%), haemorrhages (13.7%), and emesis (11.6%). These toxicities were also the ones that appeared as the most common grade I and grade II events (Table 3). Toxicities reaching grades III–IV were diarrhea, mucositis, haematological and liver toxicity, proteinuria, asthenia, emesis, pain, and colic (Table 3).

A reduction, delay, or suspension of treatment due to toxicity was needed in three of the patients with neutropenia (17.6%), in three patients with mucositis (17.6%), in the two patients with liver toxicity (100%), and in two patients with diarrhea (8.3%). There have been no deaths associated with the study treatment.

### Study treatment

The median of the cycles of bevacizumab+FOLFIRI was 11.0 (6.0–14.0). Forty-one patients (43.2%) received between 3 and 9 cycles of bevacizumab+FOLFIRI, 42 patients (44.2%) between 10 and 20 cycles, and 12 patients (12.6%) >20 cycles (12.6%). Treatment with 5-FU and irinotecan was discontinued in 89 patients (94.7%), and bevacizumab was discontinued in 88 patients (93.6%). The main causes of treatment discontinuation were progression of the disease (25.9% for 5-FU and irinotecan and 29.5% for bevacizumab) and end of planned treatment (31.8% for 5-FU, 31.8% for irinotecan, and 29.4% for bevacizumab). However, the development of adverse events was the cause of treatment discontinuation in only 7.2% of patients receiving 5-FU and irinotecan, and 8.4% of those receiving bevacizumab.

### Surgical treatment of the liver

Surgery for liver metastases was performed in 12 patients (13.8%), with a mean time from the study treatment to surgery of 5.5 weeks (range, 2.0–22.0) and curative intention in 11 patients (91.7%). The number of resected metastases ranged from 1, in five patients (41.7%), to 4, in two patients (16.7%), and a total resection was achieved in eight patients (66.7%). During the post-operative period, anastomotic leak occurred in two patients (16.7%), wound infection in one patient (8.3%), and intra-abdominal infection in two patients (16.7%). No patient was operated a second time, and there were no post-operative deaths.

## Discussion

The management of patients with mCRC has considerably changed in the last few years, mostly due to the incorporation of new agents such as the anti-VEGF antibody bevacizumab (Omura, 2008). The results from this study increase the amount of information available, and support the beneficial effect of adding bevacizumab to the FOLFIRI regimen for the treatment of mCRC. Moreover, the rate of response achieved (50.5%), the median of TTP (10.6 months), of PFS (10.6 months), and of OS (20.7 months) are in agreement with the results from clinical trials conducted so far about the combination of bevacizumab and standard regimens of irinotecan+5-FU+LV. It should be noted that the patients included in this study were not selected according to rigid criteria, and clinical trials can be extrapolated to the general population under 70 years. The administration of irinotecan, 5-FU, and LV as first-line treatment for mCRC in intravenous infusion according to the FOLFIRI regimen has shown to improve the PFS (7.6 *vs* 5.9 months; *P*=0.004) compared with the administration in bolus (IFL), although no significant differences were reached in the rate of response (47.2% *vs* 43.3%, respectively) or in the OS (23.1 *vs* 17.6 months) (Fuchs *et al*, 2007). Additionally, adding bevacizumab improved the efficacy of the treatment, prolonging the PFS to values similar to those obtained in our study (5.9 *vs* 8.3 months for IFL and IFL+bevacizumab, respectively; 7.6 *vs* 11.2 months for FOLFIRI and FOLFIRI+bevacizumab) (Fuchs *et al*, 2007). Even though there were no significant differences between the administration of FOLFIRI+bevacizumab and IFL+bevacizumab in the rate of response (57.9% *vs* 53.3%, respectively) or the PFS (11.2 *vs* 8.3 months, respectively), the OS was enlarged in patients treated with FOLFIRI+bevacizumab (*P*=0.007).

Increases in the rate of response (44.8% *vs* 34.8% (Hurwitz, 2004); 45% *vs* 35% (Popov, 2008)), smaller than those described in our study, and very similar PFS (10.6 *vs* 6.2 months (Hurwitz, 2004); 11 *vs* 6.5 months (Popov, 2008)) and OS (20.3 *vs* 15.6 months (Hurwitz, 2004); 20 *vs* 15 months (Popov, 2008)) were obtained in clinical trials that added bevacizumab to IFL regimens, representing a significant improvement compared with the group treated with IFL only. Even better results have recently been reported from a single-arm phase II trial, in which FOLFIRI+bevacizumab administration achieved a response rate of 65%, and a median PFS and OS of 12.8 and 31.3 months, respectively (Kopetz *et al*, 2010).

These results are similar or even better than those obtained with the administration of other chemotherapy combinations that are currently under study such as FOLFOX (oxaliplatin+5-FU+LV) (OR: 41% (Hochster, 2006), TTP: 8.7 months (Hochster, 2006), OS: 19.6 months (Cassidy, 2008)), CapeOx (capecitabine+oxaliplatin) (OR: 27%, TTP: 5.9 months (Hochster, 2006)), CAPIRI (capecitabine+irinotecan) (OR: 29.4%, PFS: 11.4 months, OS: 15 months (Moehler, 2009)), or XELOX (capecitabine+oxaliplatin) (PFS: 8.0 months, OS: 19.8 months (Cassidy, 2008)). Additionally, the incorporation of bevacizumab to some of these regimens has shown to significantly improve the efficacy, with an increased rate of response (FOLFOX+bevacizumab: 52% *vs* 41% CapeOx+bevacizumab: 46% *vs* 27%), TTP (FOLFOX+bevacizumab: 9.9 *vs* 8.7 months; CapeOx+bevacizumab: 10.3 *vs* 5.9 months (Hochster, 2006)), and PFS (XELOX+bevacizumab: 9.3 *vs* 7.4 months (Tyagi, 2006)). However, this improvement does not represent a substantial advantage over the regimens of bevacizumab+irinotecan+5-FU+LV. Actually, recent randomised clinical trials carried out to compare FOLFIRI+bevacizumab with other regimens containing bevacizumab as first-line treatment for mCRC have not found significant differences in efficacy. Thus, the comparison of CAPIRI+bevacizumab and FOLFIRI+bevacizumab did not find significant differences in response rates (40.7% *vs* 40.4%), median PFS (10.1 *vs* 10.5 months) or median OS (29.9 *vs* 27.9 months) (Ziras *et al*, 2010). In the same way, comparison of XELIRI (irinotecan+capecitabine)+bevacizumab and FOLFIRI+bevacizumab did not find significant variation either in response rates (OR: 38% *vs* 40% CR: 4% *vs* 3% PR: 34% *vs* 37% SD: 20% *vs* 28%) or in median PFS (14.6 *vs* 15.8 months) or OS (20.0 *vs* 26.2 months) (Pectasides *et al*, 2010). Although efficacy results of another clinical trial comparing FOLFOXIRI (oxaliplatin+5-FU+LV+irinotecan)+bevacizumab and FOLFIRI+bevacizumab are not available yet, the safety analysis of the first 100 randomised patients suggest that both treatments are safe, with a lower incidence of most grade III–IV toxicities in patients treated with FOLFIRI+bevacizumab (Falcone *et al*, 2010).

The safety of chemotherapeutic agents is other fundamental aspect of the treatment of cancer patients. However, there is currently little information available about adverse effects, clinical management, and effects on subsequent treatments in clinical practice outside of the clinical trials (Fortner, 2007). In this respect, this study provides more information based on the review of the medical charts of patients that received bevacizumab+FOLFIRI as first-line treatment. The results from our study have shown that bevacizumab+FOLFIRI combination has a good safety profile, with mostly haematologic toxicity, diarrhea, mucositis, asthenia, haemorrhages, and emesis, and, in most cases in grades I–II and only reaching grades III–IV in between 1.1% and 9.5%. This good tolerability is a key factor in identifying optimal treatment regimens and points at the bevacizumab+FOLFIRI combination as a promising candidate for CRC treatment. However, the intensity and frequency of the described adverse events does not coincide with results obtained in other clinical trials on the administration of bevacizumab+irinotecan+5-FU+LV combinations in bolus or infusion, in which higher percentages of grades III–IV adverse events were detected (Hurwitz, 2004; Fuchs *et al*, 2007; Falcone *et al*, 2010). Similar discrepancies have been previously described in other observational studies and mainly attributed to the lack of documented information in the medical charts, highlighting the need to improve the detailed information in the medical records in order to obtain a more complete source of information that will permit a greater degree of accuracy (Fortner, 2007).

In summary, the results obtained in this study have reached values for rate of response, TTP, PFS, and OS that support the beneficial effect of adding bevacizumab to the FOLFIRI regimen as first-line treatment for mCRC. The high tolerability shown by the bevacizumab+FOLFIRI regimen suggests that this combination is a promising candidate for mCRC treatment. However, the safety profile described might be also influenced by the variability in the adverse events recording in the patients' medical charts. The authors recognise that although the observational studies provide valuable information, they are not usually capable of providing strong evidence. This fact, together with the limitations derived from the retrospective collection of data from medical charts and the lack of a control group, entails the need to perform subsequent studies to confirm the results described in the present manuscript.

## Figures and Tables

**Table 1:** Patient characteristics at baseline visit (*N*=95)

**Patient characteristics**	**Value**
Median of age, years (range)	59.0 (53.2–67.1)
	
*Gender, n* (%)	
Male	61 (64.2)
Female	34 (35.8)
	
*ECOG, n* (%)	
ECOG 0	49 (51.6)
ECOG 1	43 (45.3)
ECOG 2	3 (3.2)
	
*Location of primary tumour, n* (%)^a^	
Colon	66 (69.7)
Rectum	35 (36.8)
	
*Location of metastases, n* (%)	
Liver	68 (71.6)
Lymph nodes	20 (21.1)
Peritoneum	17 (17.9)
Lung	16 (16.8)
Other	12 (12.8)
	
Median number of metastatic sites (range)	1.0 (1.0–2.0)
Prior adjuvant treatment, *n* (%)	35 (36.8)

Abbreviation: ECOG=Eastern Cooperative Oncology Group.

a Multiple response, percentages may exceed 100%.

**Table 2:** Response rates (*N*=95)

**Response rate**	***n***	**%**	**95% CI**
Complete response	8	8.4	3.7–15.9
Partial response	40	42.1	32.0–52.7
Stable disease	16	16.8	9.9–25.9
Progressive disease	31	32.6	23.4–43.0

Abbreviation: CI=confidence interval.

**Table 3:** Grades I–II and grades III–IV toxicities (*N*=95)

**Toxicity^a^**	**Grades I–II, *n* (%)**	**Grades III–IV, *n* (%)**
Haematological toxicity	34 (35.8)	9 (9.5)
Diarrhea	26 (27.3)	8 (8.5)
Mucositis	24 (25.3)	5 (5.3)
Asthenia	18 (19.0)	2 (2.1)
Emesis	10 (10.6)	1 (1.1)
Haemorrhages^b^	11 (11.6)	0 (0.0)
Pain	9 (9.7)	1 (1.1)
Nausea	9 (9.5)	0 (0.0)
Hypertension^c^	9 (9.5)	0 (0.0)
Constipation	6 (6.4)	0 (0.0)
Proteinuria	3 (3.2)	1 (1.1)
Hepatic toxicity	1 (1.1)	2 (2.1)
Alopecia	3 (3.2)	0 (0.0)
Headache	2 (2.1)	0 (0.0)
Colic	1 (1.1)	1 (1.1)
Hiccups	2 (2.1)	0 (0.0)
Thrombophlebitis	1 (1.1)	0 (0.0)
Wound healing problems	1 (1.1)	0 (0.0)
Allergic reactions	1 (1.1)	0 (0.0)
Dyspnea	1 (1.1)	0 (0.0)
Edema	1 (1.1)	0 (0.0)
Oesophagitis	1 (1.1)	0 (0.0)
Fever^c^	1 (1.1)	0 (0.0)
Neuropathy	1 (1.1)	0 (0.0)

a Infection was also reported in one patient, and lung thromboembolism and haemorrhoids in two patients, but the grade was not recorded.

b Two patients had this adverse event, but the grade was not recorded.

c One patient had this adverse event, but the grade was not recorded.
